# A Probabilistic Fragment-Based Protein Structure Prediction Algorithm

**DOI:** 10.1371/journal.pone.0038799

**Published:** 2012-07-19

**Authors:** David Simoncini, Francois Berenger, Rojan Shrestha, Kam Y. J. Zhang

**Affiliations:** 1 Zhang Initiative Research Unit, Advanced Science Institute, RIKEN, Wako, Saitama, Japan; 2 Department of Computational Biology, Graduate School of Frontier Sciences, The University of Tokyo, Kashiwa, Chiba, Japan; University of Michigan, United States of America

## Abstract

Conformational sampling is one of the bottlenecks in fragment-based protein structure prediction approaches. They generally start with a coarse-grained optimization where mainchain atoms and centroids of side chains are considered, followed by a fine-grained optimization with an all-atom representation of proteins. It is during this coarse-grained phase that fragment-based methods sample intensely the conformational space. If the native-like region is sampled more, the accuracy of the final all-atom predictions may be improved accordingly. In this work we present EdaFold, a new method for fragment-based protein structure prediction based on an Estimation of Distribution Algorithm. Fragment-based approaches build protein models by assembling short fragments from known protein structures. Whereas the probability mass functions over the fragment libraries are uniform in the usual case, we propose an algorithm that learns from previously generated decoys and steers the search toward native-like regions. A comparison with Rosetta AbInitio protocol shows that EdaFold is able to generate models with lower energies and to enhance the percentage of near-native coarse-grained decoys on a benchmark of 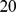 proteins. The best coarse-grained models produced by both methods were refined into all-atom models and used in molecular replacement. All atom decoys produced out of EdaFold’s decoy set reach high enough accuracy to solve the crystallographic phase problem by molecular replacement for some test proteins. EdaFold showed a higher success rate in molecular replacement when compared to Rosetta. Our study suggests that improving low resolution coarse-grained decoys allows computational methods to avoid subsequent sampling issues during all-atom refinement and to produce better all-atom models. EdaFold can be downloaded from http://www.riken.jp/zhangiru/software/.

## Introduction

Fragment based Protein Structure Prediction (PSP) algorithms have become very successful during the last decade. This could be due to the increasing number of solved structures available in the Protein Data Bank [1], and the proven efficiency of this approach. Amongst all the methods developed, Rosetta is one of the most well-known [2], along with the recently published Quark method [3]. The Rosetta protocol consists of a coarse-grained optimization phase where backbone atoms and centroids of side chains are considered, followed by a fine-grained optimization phase with a high resolution all-atom representation of proteins. The fragment assembly takes place during the first phase. Short fragments of known proteins are assembled by a Monte Carlo strategy to generate decoys. The data inside fragments are Φ, Ψ and Ω torsion angles. The torsion angles determine the backbone of proteins and replacing one fragment inside a decoy can yield important modifications of the global conformation. At this point, a protein model is only represented by a sequence of Φ, Ψ and Ω torsion angles triplets, each of them being associated with one residue of the target sequence. After fragment insertion, small random variations of Φ and Ψ angles are introduced [4]. During the fine-grained second phase, side chain conformations are optimized. Although small perturbations of main chain are also performed, they hardly affect the overall fold of the decoy.

Rosetta has been successfully used for the prediction of high resolution protein structures from sequences [4]. It has been shown that the coarse-grained conformation sampling plays an important role in predicting highly accurate structure of proteins [5]. In addition, when coarse-grained models are predicted near native structure, switching the models to all-atom representation subsequently gives solutions in Molecular Replacement trials without extensive optimization [6].

The two main challenges in PSP are on the one hand the accuracy of energy functions and on the other hand the quality of the conformational search. The conformational search space is determined by the energy function and the representation of the solutions. Despite of its imperfections, Rosetta’s all-atom energy function can distinguish native from non-native structures [7]. The challenge we are interested in is, given an accurate energy function, to increase the proportion of near native decoys during the conformational search. To achieve this goal, an optimization method must have features that can bring some knowledge on the search space. Resampling techniques and methods inspired by evolution such as genetic algorithms possess this kind of features and thus are good candidates for this task.

Resampling techniques aim at gathering information on the energy landscape from an initial round of search, and then using this information to guide the sampling of the conformational search space in subsequent rounds. A previous study suggest to project conformations onto a discrete space and to compute numerous statistics in order to identify native-like features [8]. A model-based approach using fragment assembly and Rosetta energy function has also been proposed. It has the ability to concentrate the search in regions of the search space containing extrema of the energy function [9]. Another study propose to use an iterative algorithm combining hidden Markov models and fragment assembly. The algorithm uses protein fragments to build cosine models describing residue torsion angles and a position specific hidden Markov model to capture dependencies between adjacent residues. The protein models generated during an iteration are then used to train the cosine models for the subsequent iteration [10]. Previously, the Fisher Bingham distribution has been used to parameterize the local structural preferences of residues [11]. In genetic algorithms, information is shared by recombination of good solutions. The potential of this class of algorithm in the PSP field has been discussed [12]. Probabilistic fragment selection approaches have been investigated. A previous work suggests to measure energetic fluctuations induced by local changes of the protein structure, and manage to find low energy conformations with a genetic algorithm. However, no improvement in prediction quality is reported [13]. Probabilistic models have also been used in fragment-free approaches [14], [15]. A conformational search method combining simulated annealing, genetic algorithms and molecular dynamics was reported [16]. The use of genetic algorithm was shown to reach lower energy levels than classic simulated annealing molecular dynamics algorithm. The application of Estimation of Distribution Algorithms (EDAs) to the solution of the PSP problem in simplified models has been introduced and their use as a simulation tool for the analysis of the protein folding process has been proposed [17]. The EDAs has been used in this case to model proteins in a simplified lattice HP model that separates amino acids into two classes: Hydrophobic and Polar [17]. To the best of our knowledge, there is no successful report of the use of Estimation of Distribution Algorithms on the PSP problem with atomic level representation.

In this paper we present EdaFold, a fragment based approach for protein folding using an Estimation of Distribution Algorithm [18] in order to share information between parallel simulations and enhance the search process. The proposed method tackles the coarse-grained PSP problem and could improve the quality of predicted structures for most of the tested targets compared to Rosetta. It was observed on a benchmark of 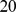 target proteins that EdaFold could generate coarse-grained decoys with significantly lower energy than Rosetta. Whereas this result showed the efficiency of the minimization process, some targets present abnormal energy/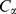 root mean square deviation (CARMSD ) to native correlation which, to a certain extent, misleads the search process. This discrepancy is caused by the quality of the low resolution energy function. On the majority of the targets, EdaFold is able to sample more efficiently near the native structure compared to Rosetta AbInitio protocol.

It has been shown recently that coarse-grained conformational sampling (where only backbone atoms are present) is the key step in *ab initio* protein folding in order to obtain models that pass the stringent test of molecular replacement [6]. Indeed, optimizing an all-atom model is a waste of computing power when the backbone conformation is far from the native structure. This study thus focuses on coarse-grained conformational sampling improvement, in order to enrich the population of generated decoys with more models near the native fold.

Rosetta’s low resolution energy function, used in coarse-grained sampling, is not as accurate as its all-atom counterpart. Often, some low energy conformations will be far from the target’s native structure. Nevertheless, it has been observed in many cases that the region containing the highest concentration of low energy conformations is located near the target’s native structure [19]. Since the majority of low energy conformations belong to the same region of the search space they share some common, native-like, structural properties. However, the size of the conformational search space leads to the design of methods that are able to sample broadly and thus are unlikely to focus on the native-like region. The goal of our approach is to enhance the search in the native-like region by defining some probability mass functions (PMFs) over the fragment library used to generate the conformations. We borrow some principles from a kind of evolutionary algorithms, the so called Estimation of Distribution Algorithms, and apply them to the PSP problem. Some features of proteins are easy to predict and will be frequently present in low energy decoys. The probability of selecting the corresponding fragments will be high. This can be exploited to design a sampling algorithm that spares useless effort on easy regions of the protein and spends more time on trying to minimize difficult ones.

Estimation of Distribution Algorithms belong to the class of Evolutionary Algorithms. In these population-based meta heuristics, solutions share some information about promising regions of the search space in order to converge toward the global optimum. While in classical evolutionary approaches newly sampled solutions are generated by explicit recombination of the population’s best solutions, EDAs strategy is to regenerate a pool of solutions with an estimated biased distribution. The distribution is biased by observation of the search sub-space described by good solutions in the population. Our strategy for the PSP problem is to alternate sampling/minimization and estimation of distribution stages. Each sampling stage will be performed with a distribution biased by the current status of the population of solutions. Premature convergence of the solutions towards a local minimum is easier to avoid than with other evolutionary approaches since one can control the influence of the population of solutions on the distribution.

## Results

In this section, we describe and analyze the results related to the sampling efficiency, both in terms of energy and CARMSD to native structure. We compare EdaFold to Rosetta AbInitio protocol, a state-of-the-art method in fragment-based protein structure prediction. EdaFold is computationally more expensive than Rosetta. It is 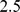 times slower than the latter on average on our protein benchmark. Even though we compare the two methods in terms of percentage of near-native decoys, we use equal computational time since the decoys generated by EdaFold are sharing information through the EDA algorithm. If EdaFold generates more models, more information will be shared and it may have an effect on the distribution of decoys. Therefore, in our experiments, we generated 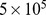 decoys for each target with Rosetta AbInitio protocol and 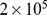 decoys with EdaFold. The two algorithms are evaluated on a set of 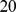 proteins from various fold families. We selected proteins from two datasets presented in previous studies [7], [8]. We removed from this benchmark proteins that seemed either too easy or too difficult to predict for Rosetta or longer than 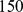 residues. We obtained a set of 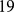 proteins, and added 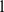 which satisfied our length and difficulty requirements to obtain a 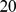 proteins benchmark.

### Energy Study

**Figure 1:**
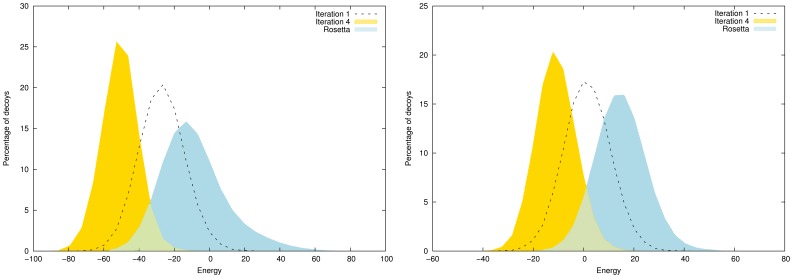
Histograms of decoys’ energy distribution: comparison between iterations 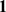, 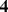 of EdaFold and Rosetta for 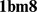 (left) and 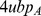 (right). The minimization process of EdaFold is more efficient than Rosetta at iteration 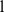. The Estimation of Distribution Algorithm allows EdaFold to increase the performances from iteration 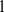 to iteration 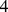.

Histograms of decoys distribution (in percentage of total number of decoys) as a function of energy are presented in Figure 1. EdaFold is able to find decoys with lower energy compared to Rosetta, and in higher proportions. For target 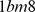, 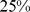 of decoys generated by the former at iteration 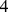 reach around 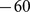 in Rosetta energy against less than 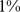 for the latter. For target 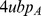, 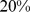 of EdaFold conformations reach around 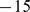 in Rosetta energy at iteration 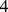 against less than 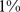 for Rosetta. Even though we only present histograms for 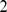 targets, the whole benchmark shows the same trend. The figure also shows the ability of the EDA to improve the energy of decoys. We note a significant improvement between iterations 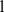 and 4. This improvement is an expected effect of the EDA implemented in EdaFold.

### Near Native Sampling Ability

One important motivation of this work is to propose an algorithm able to sample more efficiently the native-like region of the conformational space. Being able to predict near-native decoys and being able to identify them are two different issues. If a strategy cannot predict high quality decoys, then trying to find the best one is pointless. Here, we focus on improving the quantity of near-native decoys and for this reason the main results that we present are in terms of CARMSD to the native structure.

The performance of EdaFold and Rosetta is summarized in Table 1. For each target, the averages over the lowest 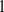 and 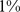 CARMSD to native are reported. We also report the average CARMSD of the lowest 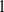 and 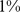 Rosetta energies. We performed a Mann-Whitney U statistical test [20] on each sample. Samples that are statistically different with a confidence higher than 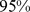 are in bold. EdaFold outperforms Rosetta AbInitio protocol on a majority of the targets in terms of near-native region sampling efficiency. Even if Rosetta’s coarse-grained energy is noisy, EdaFold still takes advantage of it and achieves a better average 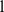 and 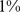 CARMSD when decoys are sorted by Rosetta energy as shown in columns *avg e- CARMSD*. EdaFold finds lower energies on all proteins in the benchmark and this results shows that this improvement is followed by a lower average CARMSD to native on a majority of targets.

**Table 1:** Performance of EdaFold and Rosetta.

		EdaFold				Rosetta			
		avg CARMSD (Å )		avg e- CARMSD (Å )		avg CARMSD (Å )		avg e- CARMSD (Å )	
Target	Length	top 1	top 1%	top 1	top 1%	top 1	top 1%	top 1	top 1%
1*bq*9	54	**1.98**	**2.97**	9.13	8.87	3.53	4.63	9.00	7.83
1*di*2	69	**1.35**	**1.57**	4.23	4.75	1.51	1.91	4.17	**4.07**
1*scj_B_*	71	**2.66**	**3.05**	**3.08**	**3.06**	3.62	4.22	4.60	4.42
1*hz*5	72	2.28	2.6	3.95	4.06	**2.23**	**2.49**	**3.88**	**3.80**
1*cc*8	73	**2.69**	3.3	6.80	6.42	2.71	**3.22**	**5.05**	**3.60**
1*ctf*	74	3.19	3.94	**7.09**	**7.05**	**3.1**	**3.73**	8.37	7.92
1*ig*5	75	2.34	2.75	**4.55**	4.16	2.34	**2.72**	5.03	4.30
1*dtj*	76	2.73	**3.66**	6.82	5.85	2.73	3.7	**6.29**	**3.58**
1*ogw*	76	**2.64**	**3.07**	**4.66**	4.66	2.89	3.29	4.70	**4.61**
1*dcj*	81	**2.76**	**3.41**	**5.18**	**5.00**	2.91	3.52	5.99	5.06
2*ci*2	83	3.23	4.72	**8.10**	**8.12**	**3.16**	**4.17**	9.15	9.95
3*nzl*	83	**3.74**	**4.14**	**5.26**	**5.27**	3.94	4.49	7.33	7.75
1*a*19	90	**3.18**	**3.76**	**5.55**	**4.44**	3.46	4.37	8.62	8.52
1*tig*	94	3.4	**3.83**	4.96	4.52	**3.33**	3.95	**4.75**	4.13
1*bm*8	99	3.67	**4.36**	8.89	**6.71**	3.65	4.57	8.82	8.73
4*ubp_A_*	101	4.13	4.87	11.24	12.50	**3.85**	**4.63**	**9.87**	**8.51**
1*iib*	106	3.29	**4.42**	**9.41**	**9.72**	3.28	4.7	10.44	11.28
1*m*6*t*	106	**1.67**	**2.01**	**3.56**	4.82	1.94	2.34	3.98	**3.51**
1*acf*	125	**3.96**	**4.68**	10.40	**8.54**	4.75	5.92	10.34	12.17
3*chy*	128	**3.52**	**4.51**	**6.99**	**4.87**	3.88	4.93	7.81	5.90
Avg.		**2.92**	**3.58**	**6.49**	**6.17**	3.14	3.88	6.90	6.48

*avg CARMSD* is the average CARMSD of the nearest to native decoys. *avg e- CARMSD* is the average CARMSD of the lowest Rosetta energy decoys. Results differences which are statistically significant with more than 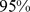 confidence according to Mann-Whitney U test are in bold.

**Figure 2:**
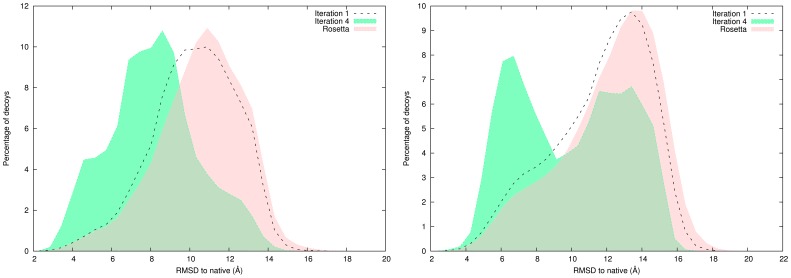
Histogram of CARMSD to native decoy distribution: comparison between iterations 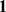, 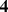 of EdaFold and Rosetta for 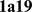 (left) and 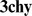 (right). EdaFold is able to guide the sampling towards native structure: the percentage of near-native models is higher at iteration 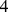.

A histogram of the distribution of predicted decoys with EdaFold and Rosetta as a function of CARMSD to native for the targets 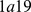 and 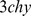 is shown in Figure 2. Iterations 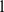 and 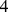 of EdaFold are displayed. Histograms of EdaFold ‘s iteration 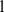 have the same shape as the ones from Rosetta. At iteration 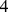, the distributions drift toward the native structure. Looking at Rosetta histograms, the highest peak in the distribution is away from the native structure in both cases. The search dynamic is different from one target to the other. The distribution becomes clearly bimodal at iteration 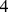 for target 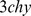. The benefit of sharing information between parallel processes during the search is put in evidence in these figures. CARMSD to native for all decoys were computed using the ‘ranker’ tool provided with Durandal [21]. ‘ranker’ executes in constant space and thus can handle any number of decoys.

**Figure 3:**
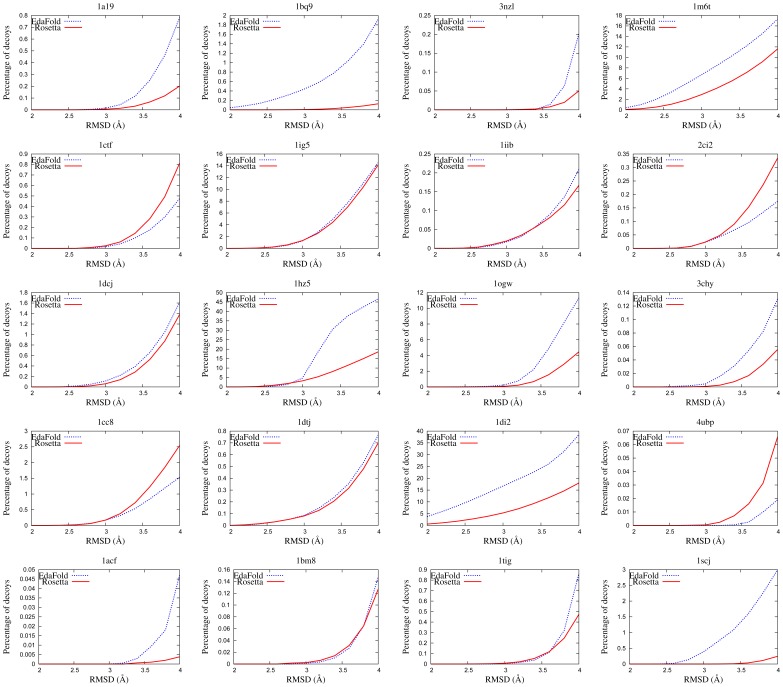
Decoys distribution as a function of CARMSD to native structure. EdaFold (in blue) is able to generate a higher percentage of decoys at less than 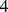 Å from native on 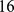 targets out of 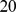.

The percentage of decoys generated with EdaFold and Rosetta that are less than a certain threshold distance from the native structure is plotted in Figure 3. The abcisse axis, representing the threshold values, ranges from 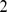 to 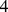 Å. The threshold values increase by a step of 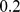 Å. If we consider that conformations less than 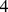 Å CARMSD away from the native structure are exploitable for subsequent all-atom refinement, then this figure gives a good picture of the ability of both methods to generate useful conformations. It appears that EdaFold is superior from this point of view on a majority of targets.

**Figure 4:**
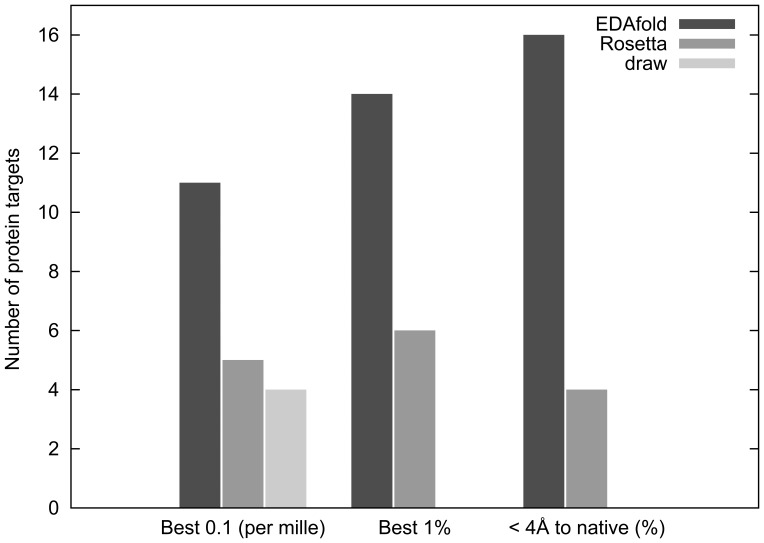
Histograms for 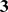 performance criteria: best 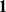 and 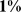 average CARMSD to native and best percentage of models generated at less than 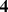 Å away from the native structure.

**Figure 5:**
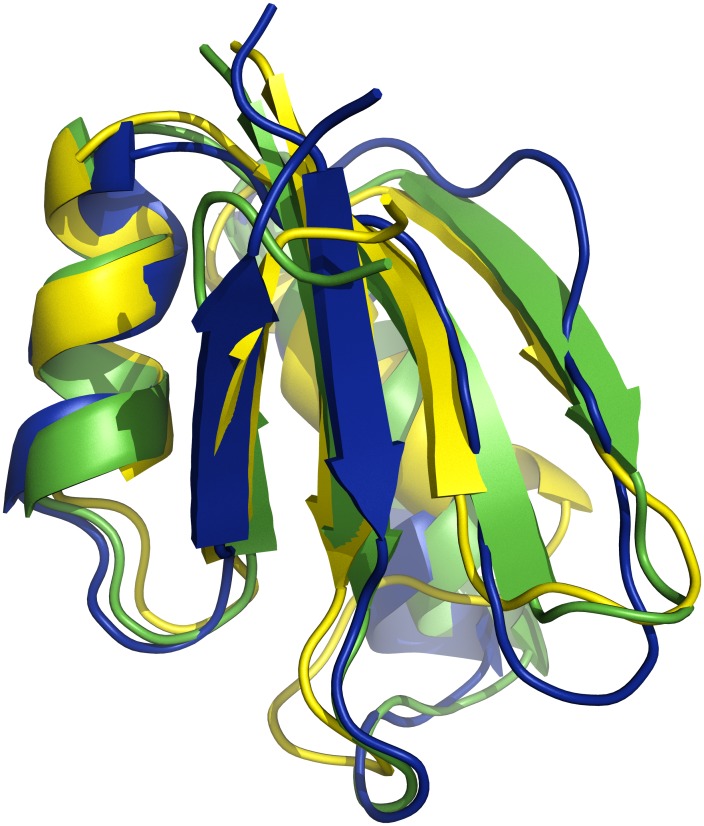
1scj models: EdaFold’s best model (in green), 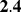 Å away from native (in yellow), Rosetta’s best model (in blue), 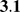 Å away from native.

A summary of the performance comparison of EdaFold and Rosetta from 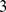 performance criteria is given in Figure 4. EdaFold outperforms Rosetta on a majority of targets either in terms of best 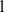 and 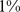 average CARMSD to native. The third criterion is extracted from Figure 3. It compares the percentage of models at less than 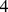 Å away from native generated by each method and the higher the better. Looking at this criterion, EdaFold is superior on 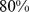 of the benchmark targets. Finally, the best models generated with EdaFold and Rosetta for target 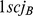 are shown in Figure 5. The models are superimposed with the native structure. The model generated by EdaFold accurately predicts 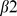 (on the right) whereas Rosetta’s one misses it. It also slightly better predicts *α*1 (in the back on the right) and 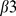. Both methods can predict 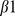, *α*2 and 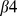.

### All Atom Models

We showed that, on a benchmark of 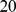 proteins, EdaFold is able to reach lower coarse-grained energies than Rosetta and to enhance the percentage of near-native decoys on a majority of targets. Here, we show that these improved low resolution coarse-grained decoys can lead to more accurate high resolution models after all-atom refinement. For 10 protein targets, we selected the 5 lowest CARMSD decoys from EdaFold and Rosetta Abinitio. For each decoy, we ran 100 Rosetta fast Relax protocol to produce 500 high resolution models for each target with both methods. We used Phaser [22] to run molecular replacement on this dataset. Molecular replacement is a stringent test to assess the accuracy of computationally generated models [23]. The goal of this experiment was to test whether improved coarse-grained models can lead to improved all-atom models after refinement. Therefore, we selected the best models of both methods by CARMSD. This won’t be possible in blind test where the native structure is not available. First, we want to separate the model generation from model selection. If a method cannot produce good enough models, then blind selection will always fail. Secondly, the blind selection of best coarse-grained decoys is moot if all the coarse-grained decoys were used for all-atom refinement. This is a matter of computational resources.

We selected proteins from our set that were also tested for molecular replacement by Rosetta [23]. We added 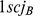 to this dataset in order to check whether low resolution models showing significant improvement in CARMSD to native could tranlate into high resolution models with the same CARMSD gap. Significant CARMSD to native improvements were observed for two proteins of our benchmark: 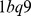 and 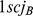. Results of this experiment are reported in table 2. Out of the 10 targets tested, EdaFold succeeded in 5 targets as compared to 3 targets succeeded by Rosetta. The success in molecular replacement is not only judged by the TFZ score but also assessed by a verification procedure [6]. For 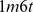, EdaFold and Rosetta manage to find a solution in molecular replacement. Both methods produced good low resolution models which led to all-atom models accurate enough for molecular replacement. For 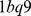, EdaFold produced better low resolution models. The corresponding all-atom models gave a solution for molecular replacement with a satisfying TFZ score, whereas Rosetta models failed the test. In the case of 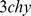, EdaFold produced slightly better low resolution models which resulted in slightly better all-atom models. Even though both methods gave a solution for molecular replacement, the TFZ score of EdaFold ‘s model (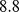) leaves no doubt about the solution whereas the TFZ score of Rosetta’s model (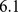) is in the grey zone and would not give confidence in solution without manual inspection or additional means of validation. In the case of 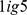, even though the best low resolution models were equally distant from native for both methods, better all-atom model were produced out of EdaFold’s decoy set which was successful in molecular replacement. For Rosetta’s decoys, no improvement was achieved after all-atom refinement and no decoy could pass the molecular replacement test. For 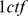, the all-atom refinement stage not only didn’t improve the CARMSD of the best coarse-grained decoys on Rosetta decoy set, but also decreased it by 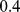 Å on EdaFold decoy set. None of the methods were successful in molecular replacement. This uncertainty in the quality of all-atom models produced by Rosetta fast Relax protocol put in evidence the necessity of enhancing the number of coarse-grained near-native decoys. If more near-native coarse-grained decoys were available, the chances that Rosetta fast Relax protocol improves one of them would be increased. Both methods failed on targets 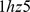, 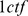, 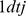, 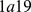 and 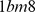. In the case of 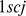, the diffraction data contains chains 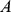 and 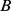. Since we only tried to predict the structure for chain 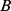, we considered that chain 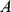 was already solved when running Phaser. Both methods obtained high TFZ scores, but during molecular replacement verification it appeared that Rosetta’s best model had a translation/symmetry CARMSD (v- CARMSD ) of 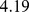 Å. In comparison, EdaFold’s best model has an v- CARMSD of 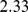 Å. CARMSD results show that even if Rosetta’s all-atom energy is not adapted for 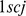, and native structure has a higher energy than incorrect models [7], this issue can be overcome by a more efficient search during coarse-grained sampling.

**Table 2:** Molecular replacement with Phaser.

Target	EdaFold						
	Best TFZ model				BestCARMSD		Best CG-CARMSD
	TFZ	v- CARMSD	CARMSD				
1*bq*9	8.0	1.15	1.08		1.04		1.30
1*scj_B_*	18.1	2.33	2.17		1.9		2.38
1*hz*5	6.0	23.3	2.63		1.8		2.01
1*ctf*	5.6	28.07	3.3		2.76		2.35
1*ig*5	7.0	1.85	1.7		1.52		1.84
1*dtj*	6.1	23.8	1.8		1.65		1.76
1*a*19	4.7	18.05	2.1		1.86		2.55
1*bm*8	5.8	17.0	3.4		2.5		2.81
1*m*6*t*	8.9	1.21	1.17		1.06		1.35
3*chy*	8.8	2.19	2.13		2.12		2.7
**Target**	**Rosetta**						
	**Best TFZ model**			**Best** **CARMSD**		**Best CG-** **CARMSD**	
	**TFZ**	**v- CARMSD**	**CARMSD**				
1*bq*9	5.3	16.73	3.33	2.55		2.97	
1*scj_B_*	10.4	4.19	3.5	2.78		3.1	
1*hz*5	6.2	23.6	2.44	1.62		1.9	
1*ctf*	5.6	19.0	3.06	2.3		2.39	
1*ig*5	5.9	20.22	2.58	1.84		1.85	
1*dtj*	5.8	21.8	1.67	1.4		1.82	
1*a*19	5.0	19.9	2.84	1.96		2.58	
1*bm*8	5.8	22.9	4.6	1.98		2.61	
1*m*6*t*	8.0	1.33	1.23	0.96		1.54	
3*chy*	6.1	2.53	2.38	2.31		2.78	

Columns labelled *Best TFZ model* show measures on the predicted model with the best Phaser TFZ score. *TFZ* stands for Translation Function Z score, typically used to determine the probability of success of a molecular replacement test. *v- CARMSD* for molecular replacement verification CARMSD, represents the CARMSD of the molecular replacement model to the native structure after optimal unit cell translation and symmetric expansion of atoms in the space group. If this value is less than 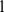
Å away from the value of the CARMSD to native after optimal superimposition by rigid body alignment (column CARMSD ) then the molecular replacement solution is considered successful. *Best CARMSD* shows the lowest CARMSD to native of the all-atom refined models used in molecular replacement. *Best CG- CARMSD* shows the lowest CARMSD found in the coarse-grained models.

## Discussion

We compared the coarse-grained sampling performances of EdaFold and Rosetta on a benchmark of 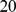 proteins. In this section, we analyze the results and discuss the benefits and shortcomings of this new method. We also suggest some leads for further improvements of EdaFold and fragment-based approaches.

### Coarse-grained Energy Function Accuracy

**Figure 6:**
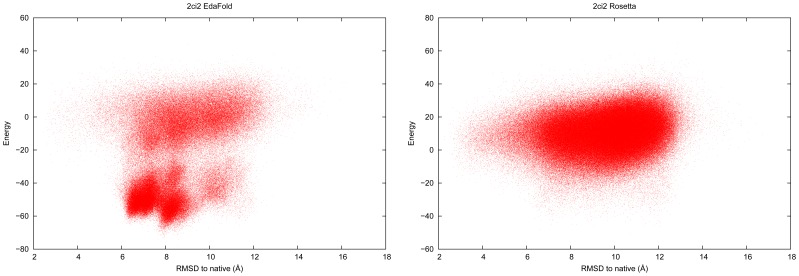
Deceptive landscape: energy as a function of CARMSD to native structure on target 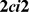 for EdaFold and Rosetta.

**Figure 7:**
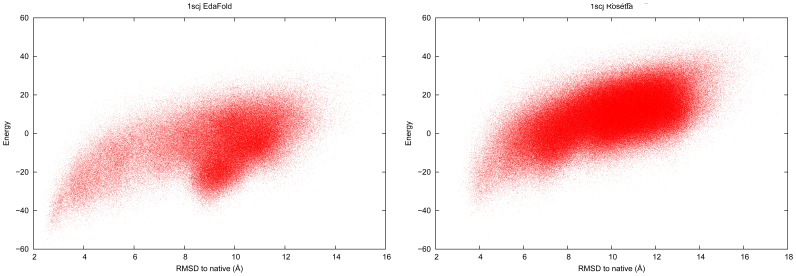
Suitable landscape: energy as a function of CARMSD to native structure on target 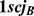 for EdaFold and Rosetta.

EdaFold relies on the energy function to densify the search in some regions of the landscape. If the energy function is not adapted for some targets, then the densification may be achieved in regions far away from the native structure. Such inaccuracies in Rosetta’s coarse-grained energy function has been pointed out in previous work [24]. Among all of the targets of our benchmark, some presented such deceptive fitness landscapes: all of the ones for which Rosetta found a higher rate of near-native conformations. In Figure 6 we plot the energy of predicted decoys as a function of CARMSD to native structure on target 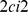 for both EdaFold and Rosetta. This figure shows that for the two methods, the lowest energies are reached for conformations which stand at around 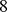 Å away from native. In the case of EdaFold, the deceptive nature of the landscape is more pronounced because it enhanced the search in this wrong region and thus discovered even lower energy decoys within the same area. If one looks at the numbers in table 1, we can see that the negative impact of such landscapes on performance is limited. One reason for this is that each iteration of EdaFold contribute equally to the total number of predicted models and the distribution used to generate models during the first iteration is uniform (see Methods section). Since we perform 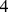 iterations for each experiment, 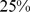 of the models (generated at iteration 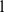) are independent. This process mitigates the loss of diversity in the final population and avoid from drifting too far from native structure in case of deceptive landscape. We note that 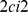 is also difficult for Rosetta, which can only generate 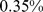 of the decoys at less than 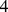 Å away from native. This issue can be solved by using a more sophisticated energy function or at least by finding a better balance between the energy terms. We could also change the adaptation policy of the EDA so that it is less sensitive to energy variations but in this case we would be less competitive on the majority of targets for which the energy function is well adapted. As an example of such target, Figure 7 shows the same energy versus CARMSD to native plot for 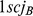. With this kind of landscape, EdaFold’s abillity to find low energy models allows to discover high quality decoys that cannot be found by Rosetta. A previous study shows that 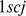 falls into the category of proteins for which incorrect non-native models reach lower (all-atom) energy levels than native structure [7]. Our study suggests that in such cases, improving the quality of low resolution models allow to overcome this issue.

### EDA-based Conformation Search Efficiency

Fragment-based approaches to the PSP problem are attractive since they allow to discretize the conformational search space and introduce some knowledge from subsequences of proteins taken from the Protein Data Bank. Our study focuses on the first part of the search process, that is the coarse-grained sampling. This part is critical because it determines the global structure of the protein models. Rosetta, as well as EdaFold propose some parallel implementation. This work shows that a negligible amount of communications between jobs executed simultaneously increases the percentage of decoys with structures close to the native during coarse-grained sampling. The benefits are twofold: first, it will translate into more successful high resolution all-atom refinement and second, it will increase the probability of near-native decoys’ blind selection. Preliminary results on all-atom refinement of EdaFold decoys are promising: the lowest CARMSD to native coarse-grained decoys translated into accurate all-atom models which could pass the molecular replacement test and solve the crystallographic phase problem for some proteins for which Rosetta protocol is unsuccessful. Although we selected the decoys with apriori knowledge of native structure, the success of molecular replacement without this information is only a computational issue which scales linearly. The core of EdaFold, which is the estimation of PMFs on the protein fragments is not specific to our minimization algorithm and can be implemented into any fragment-based approach.

EdaFold is able to consistently find lower energies than Rosetta, even when the CARMSD to native are of the same order. There are two reasons for this behaviour. First, our algorithm spends more time during the minimization phase. We systematically perform an iterated local search step after the simulated annealing sampling which is highly efficient for energy minimization. Second, it is the essence of any evolutionary algorithm to converge towards low energy regions of the landscape. Figure 1 gives an insight on the evolution of the energy as a function of iterations in EdaFold on two targets. At iteration 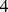, the percentage of low energy decoys is higher and the algorithm is able to find decoys with energy levels unreachable at iteration 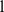. It is also clear from this figure that the minimization process in EdaFold is more efficient than Rosetta’s one, even at iteration 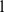. The same trend is observed on all targets of the benchmark.

Our method differs from other existing resampling techniques such as the one presented by Blum *et al.* [8] in several aspects. Since it only relies on energy in order to guide the search, it is easier to implement and any improvement of the energy function will have some direct impact on the results. Also, whereas resampling techniques perform one “control” sampling stage and one “resampling” stage, EdaFold is an evolutionary algorithm: the PMFs estimation process is iterative and refined at each evolutionary step. Finally, from an implementation point of view, the originality of our method resides in the fact that we do not need to modify the fragment libraries in order to bias the selection of fragments. We use a selection operator based on the roulette wheel algorithm which allows to pick fragments according to predefined PMFs. The Fragment-HMM method [10] is similar to our approach in the sense that it is also relying on an iterative process in order to estimate distributions over torsion angles. However, the two algorithms differ in several points. The main difference is in the estimation process. Whereas the Fragment-HMM builds cosine von Mises models and thus assume that the torsion angles follow such a distribution, EdaFold directly estimates each fragment’s probability of belonging to the correct fold. Moreover, Fragment-HMM does not use fragments as building blocks. It uses fragments to estimate the cosine models in a pre-sampling step and then uses the cosine models in an hidden Markov model during the sampling. Finally, Fragment-HMM iterates until convergence whereas EdaFold produces numerous protein models, as we consider final model selection as a post-processing operation.

## Methods

Our strategy is described in details as follows. The corner stone of the proposed algorithm is the estimated probability mass functions (PMFs) defined over the protein fragment library. Formally, 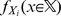 is the probability mass function for residue 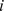, where 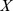 is a random variable, 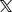 is the set 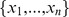 of all the values that 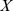 can take and 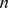 is the number of fragments involving residue 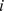. We define a probability mass function for each residue in the target sequence. At the beginning, no information on the fitness of fragments to the sequence is available and thus every fragment has the same probability of being inserted into a solution. The PMF estimations are then refined after each sample and minimization process. An outline of our search strategy is given in algorithm 1.
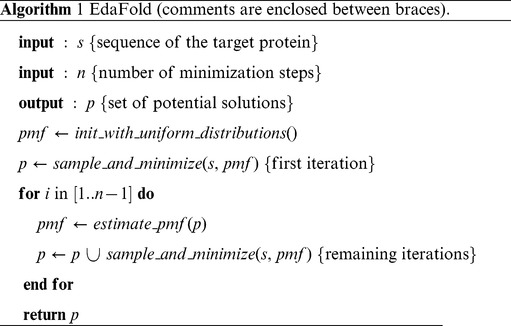


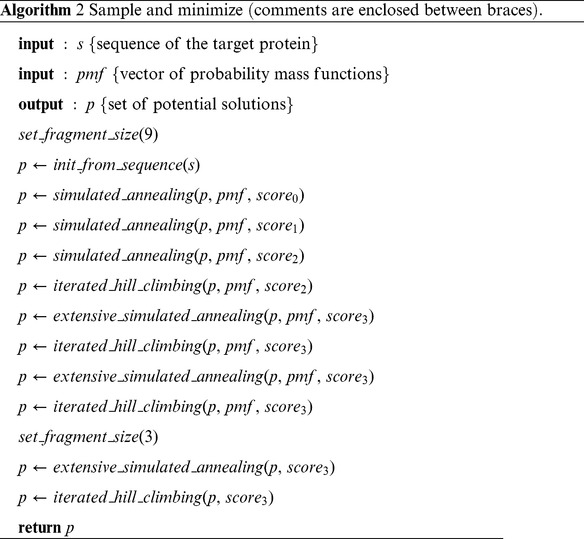



The initialization stage consists of random insertion of fragments into solutions for each residue selected in random order. The sample and minimize function is a combination of simulated annealing and iterated hill climbing. It uses Rosetta’s energy function with Rosetta scores 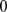, 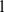, 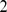 and 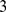. Simulated annealing alone is used with scores 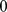 and 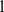. Iterated hill climbing is performed after simulated annealing with scores 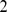 and 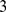. With score 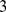, two steps of sample and minimization are performed, during which there are two calls to the simulated annealing function followed by one call to the iterated hill climbing function. The iterated hill climbing is an iterated local search algorithm [25]. It consists of an alternance of local search minimization and random perturbation of the solution. The rationale is that doing a small perturbation from a local minimum and starting again a local search is more efficient than doing random restarts of local searches. We combine this strategy with simulated annealing in order to increase the chances of escaping from local minima. The hill climbing is not exhaustive. It uses the estimated PMFs to insert fragments into the solutions, as in the simulated annealing. One iteration of hill climbing works as follows: first randomly select with no redraw an insertion window in the solution; then try to insert a fragment that decreases the energy and repeat until all insertion windows have been selected. We iterate the main loop until the desired number of iterations is reached. At each iteration, the PMFs are estimated with the following formula :
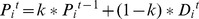
where 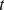 is the current iteration, 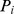 the probability of fragment 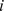, 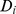 the observed frequency of fragment 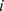 and 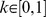 a conservation rate. In order to obtain the set of 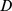, we sort the decoys by energy and compute the observed frequency of fragments on the top 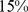. Even though 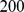 fragments are available for each fragment window of the sequence, we initially pick fragments from the top 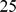 positions in the library for computational cost reasons. The algorithm is then allowed to insert any fragment in the iterated hill climbing random perturbation function. At each iteration, the population of solutions is re-initialized from the sequence. Note that the solutions used to estimate the distribution are not lost and are also stored in the final pool of solutions. An equal fraction of the final pool is generated at each iteration of the algorithm. Details of the *sample and minimize* procedure are shown in algorithm 2. The number of simulated annealing moves doubles in the *extensive* version. We allow more moves to increase chances of escaping local minima once the Rosetta energy function reaches its final coarse-grained form.

The estimation of PMFs requires to be able to trace back the angles describing the position of each residue and to know from which fragment it was taken. To do so, we assign an identifier to each fragment. Each identifier is unique relative to a residue number: for a given residue, every possible fragment has a unique identifier. We define a fragment key as :
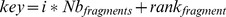
where 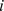 is the position of the residue in the insertion window, 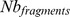 the number of fragments per window in the library and 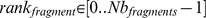 the rank of the fragment in the library. The reverse operation is:



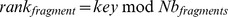


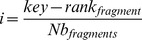



The size of the insertion windows must be constant in order for these keys to be unique. In our implementation, we estimate the probabilities on the fragments of size 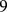. The main reason for this choice is that using uniform distributions with fragments of size 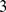 provides another way to maintain diversity in the solution set.

The parameters used in our experiments are reported in table 3. Fragments used during the experiments were generated through the Robetta Server [26]. We generated the fragments with the web interface option for excluding homologs turned on. We then checked with a local software that no proteins with more than 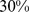 sequence identity with the target were used to generate the fragment libraries. 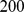 fragments of length 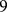 and 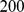 fragments of size 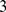 were generated for each insertion window. The same set of fragments was used for EdaFold and Rosetta predictions.

**Table 3:** EdaFold parameter settings during experiments.

General settings	
Size of Population (per iteration)	5.10^4^
Number of Iterations	4
**Probability mass function settings**	
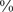 of Population	15
Conservation Rate	0.6
**Simulated annealing settings**	
Number of Steps (extensive)	4.10^3^
Initial Temperature	3.5
Final Temperature	0.5
**Iterated hill climbing settings**	
Number of Iterations	3
