# Correction: A Probabilistic Fragment-Based Protein Structure Prediction Algorithm

**DOI:** 10.1371/annotation/1131431f-8b78-4fdf-8c35-a48bde1cf1f1

**Published:** 2012-10-05

**Authors:** David Simoncini, Francois Berenger, Rojan Shrestha, Kam Y. J. Zhang

Due to a formatting error, the “permille” symbol (‰) was omitted whenever it was used in the text. Below are the locations of the errors:

1. On Page 3, left column, last paragraph, “lowest 1” should be “lowest 1‰”:

1a. “For each target, the averages over the lowest 1‰ and 1% CARMSD to native are reported.”

1b. “We also report the average CARMSD of the lowest 1‰ and 1% Rosetta energies.”

2. On Page 3, left column, last paragraph, “average 1” should be “average 1‰”:

2a. “Even if Rosetta’s coarse-grained energy is noisy, EdaFold still takes advantage of it and achieves a better average 1‰ and 1% CARMSD”

3. On Page 3, right column, last paragraph, “best 1” should be “best 1‰”:

3a. “EdaFold outperforms Rosetta on a majority of targets either in terms of best 1‰ and 1% average CARMSD to native”

4. On Page 4, table 1, “top 1” should be “top 1‰” in the row that starts with “Target”.

5. On Page 6, legend of figure 4, “best 1” should be “best 1‰”.

There was also an error in the last sentence of the Abstract. The correct link is:

http://www.riken.jp/zhangiru/software.html [^] 

